# Supplementary material for: Integration of Phenotypes in Microbiome Networks for Designing Synthetic Communities: a Study of Mycobiomes in the Grafted Tomato System
Source: Appl Environ Microbiol. 2023 May 24;89(6):e01843-22. doi: 10.1128/aem.01843-22 (PMC10304797; doi:10.1128/aem.01843-22)
Supplement: Supplemental file 1 — Supplemental material. Download aem.01843-22-s0001.pdf, PDF file, 3.6 MB [file aem.01843-22-s0001.pdf]

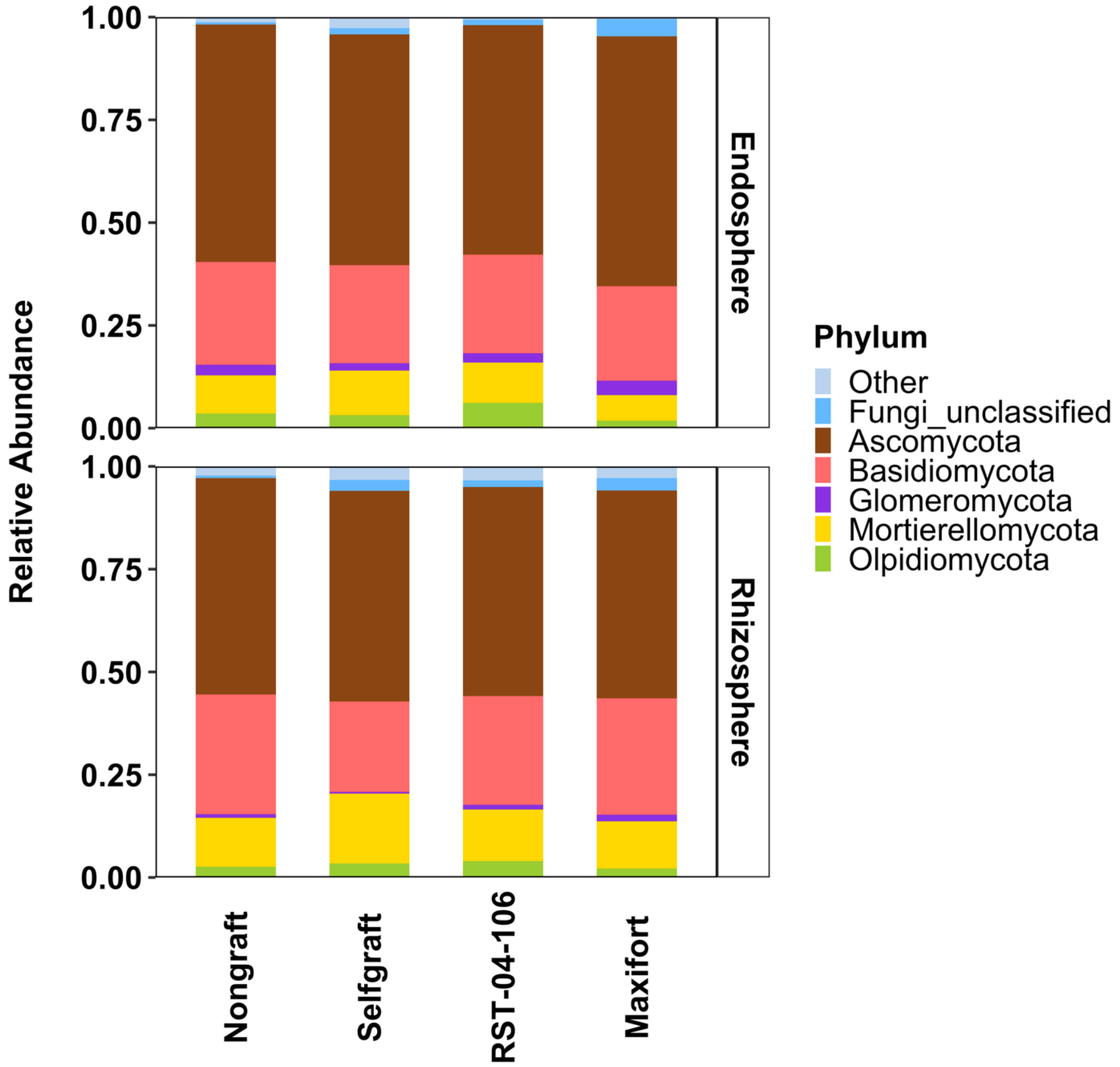

FIG S1 Relative abundance of endosphere and rhizosphere fungi at the phylum level recovered from four tomato rootstock treatments: nongraft BHN589, selfgraft BHN589, and BHN589 grafted on two hybrid rootstocks (RST-04-106 and Maxifort). Each individual bar represents a rootstock treatment, and the colored area within the bar represents the relative abundance of the corresponding phylum. Phyla with less than 1% relative abundance are labeled as ‘other’.

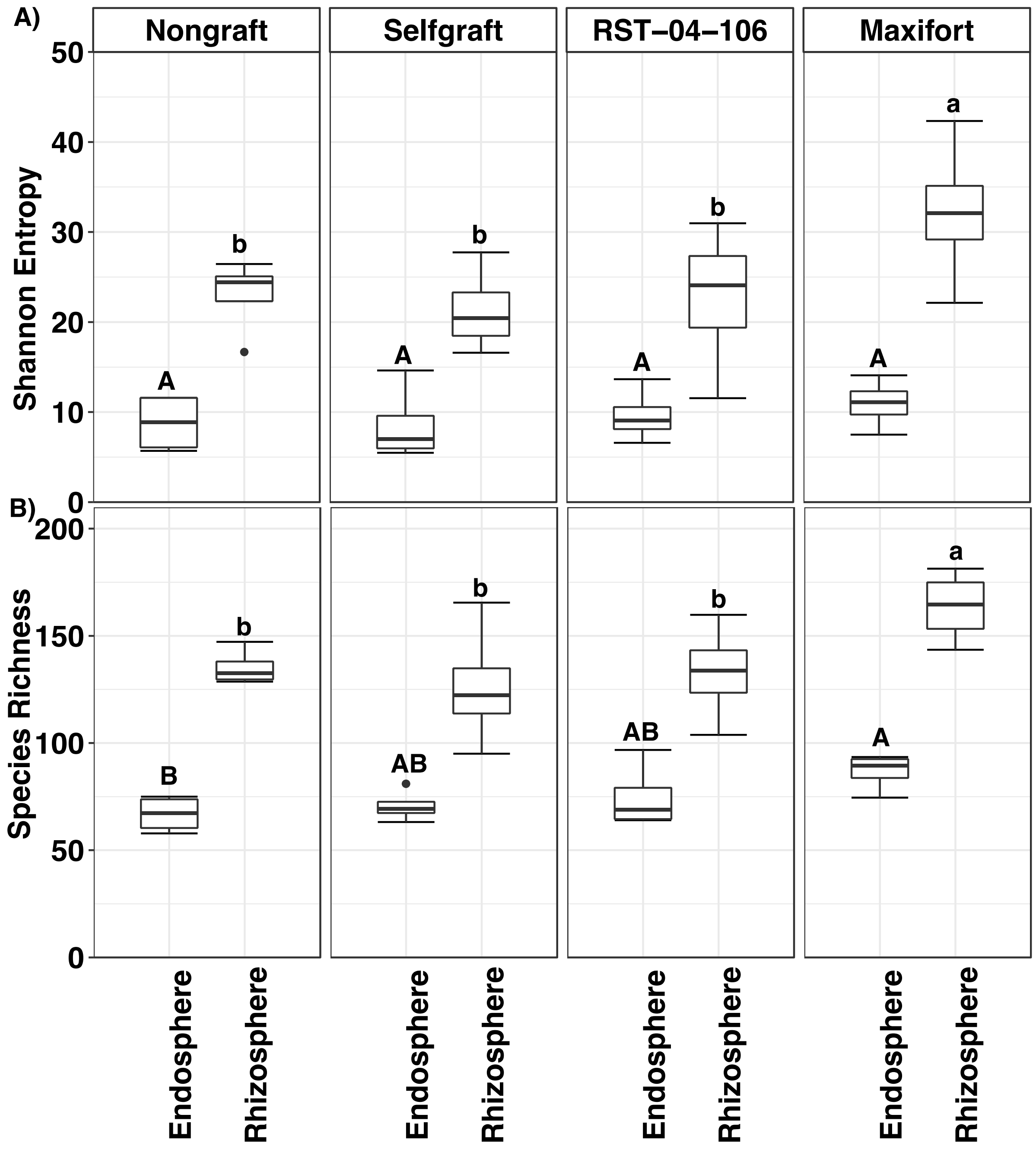

FIG S2 Comparison of overall fungal diversity (A) and richness (B) associated with tomato rootstock genotypes and controls, evaluated in the endosphere and rhizosphere. The plot is divided by the four tomato rootstock treatments: nongraft BHN589, selfgraft BHN589, and BHN589 grafted on two hybrid rootstocks (RST-04-106 and Maxifort). Shannon entropy and

species richness, measures of community diversity, were both higher for Maxifort ($p < 0.005$) compared to the self-graft and RST-04-106 in the rhizosphere, while there was no evidence for a difference in Shannon entropy in the endosphere ($p = 0.634$). Treatment means were separated using the "difflsmeans" function as specified in the lmerTest package in R. Tests for boxplots sharing a letter or letter case type had $p > 0.05$.

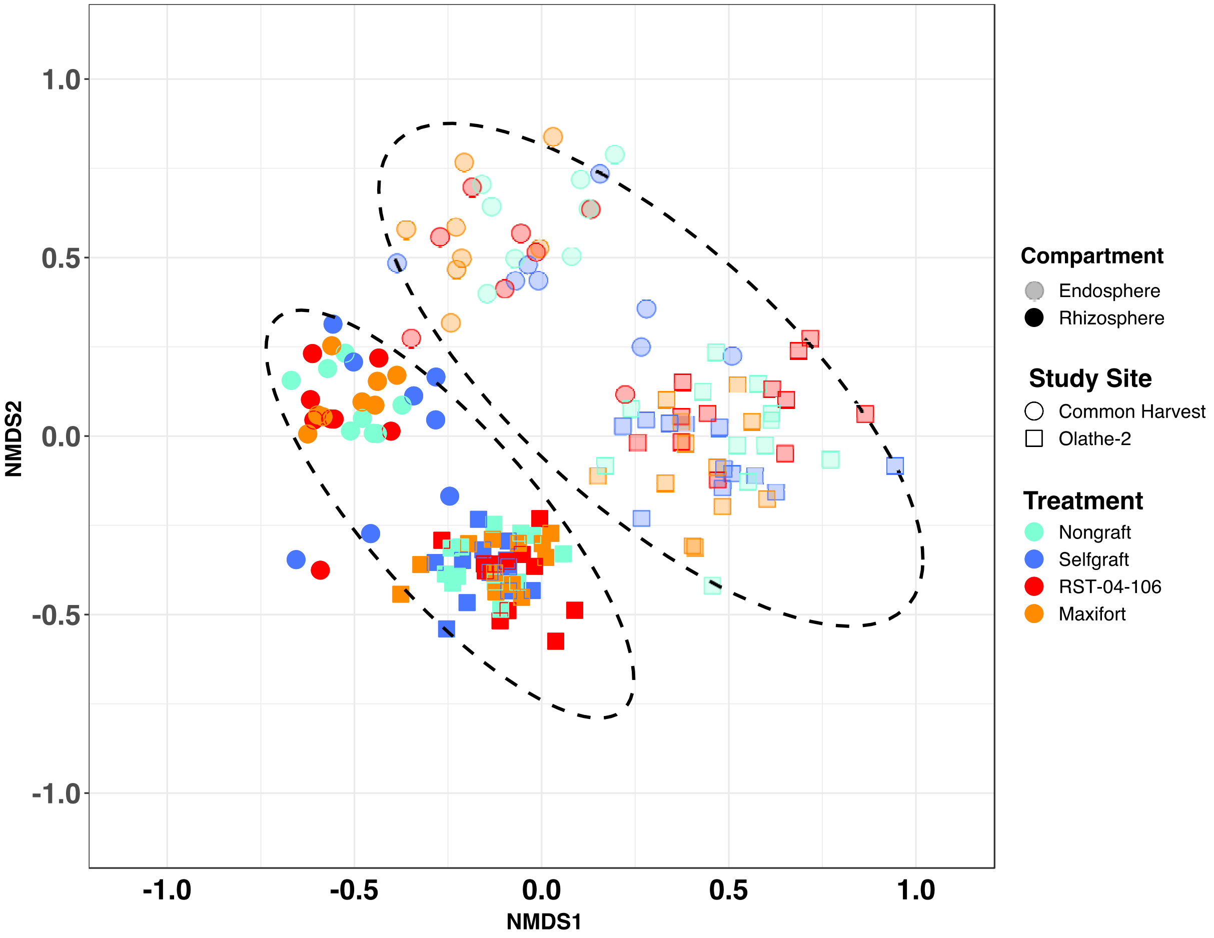

FIG S3 Non-metric multidimensional scaling (NMDS) ordination plot of samples labeled by tomato rootstock (nongraft BHN589, selfgraft BHN589, and BHN589 grafted on two hybrid rootstocks (RST-04-106 and Maxifort)), compartment (endosphere or rhizosphere), and study site, based on the Bray-Curtis dissimilarity distance matrix of fungal OTUs. Color indicates rootstock treatment, shape indicates study site, and size indicates compartment. Ellipses surrounding the samples indicate the 95% CI of the endosphere and rhizosphere sample centroids.

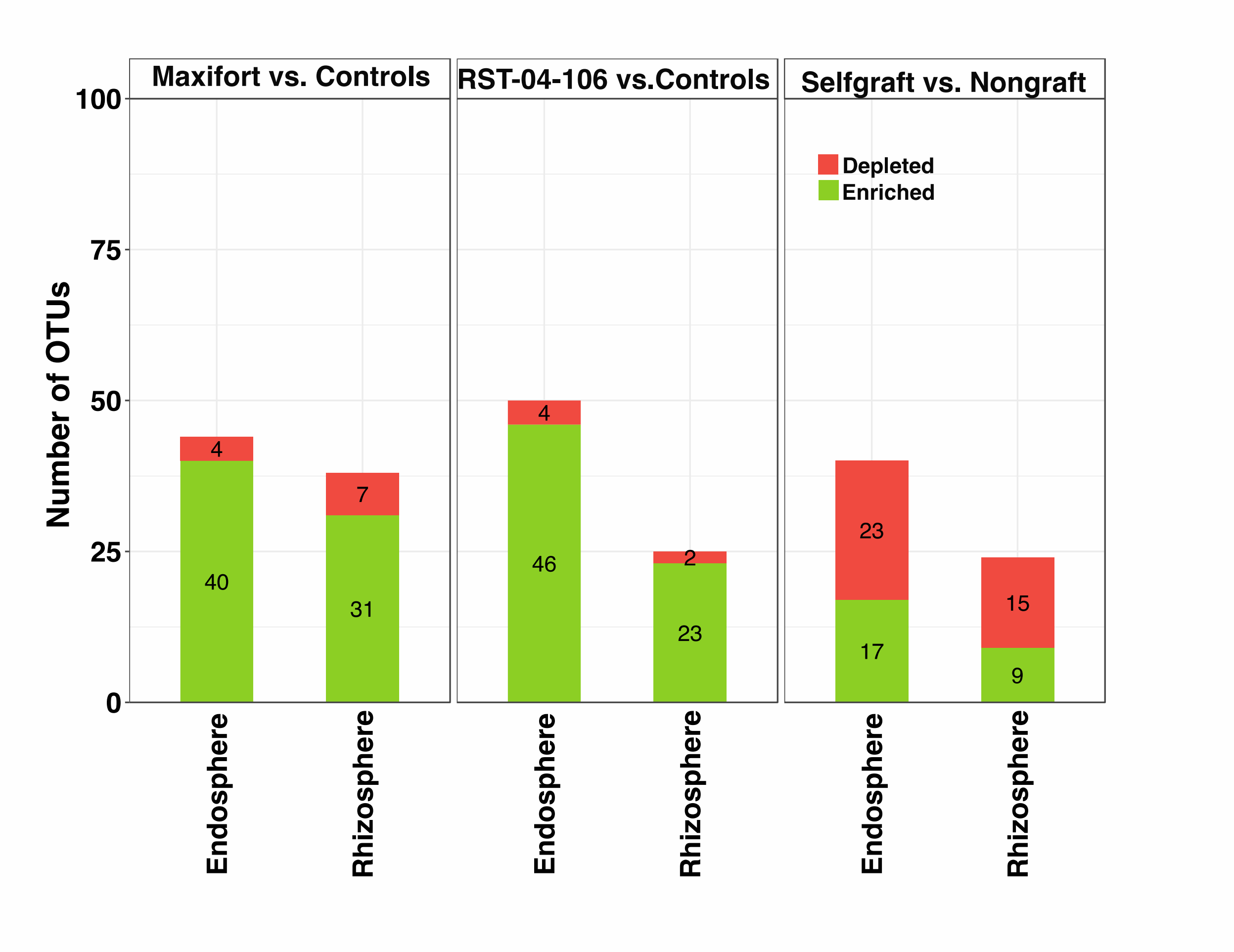

FIG S4 Number of DAOTUs in a contrast analysis, evaluated for the endosphere and rhizosphere compartments for four tomato rootstock treatments: nongraft and selfgraft BHN589, and BHN589 grafted on two hybrid rootstocks (Maxifort and RST-04-106). The green color in each bar represents the number of enriched taxa, and the red color represents the number of depleted taxa. The number of differentially changed taxa was greater for the endosphere than for the rhizosphere. Among the contrast pairs, hybrid rootstocks had a greater number of enriched taxa compared to depleted taxa. However, the number of depleted taxa was higher compared to enriched taxa in the controls. Among the treatments, Maxifort had the highest number of DAOTUs in both compartments.

A)

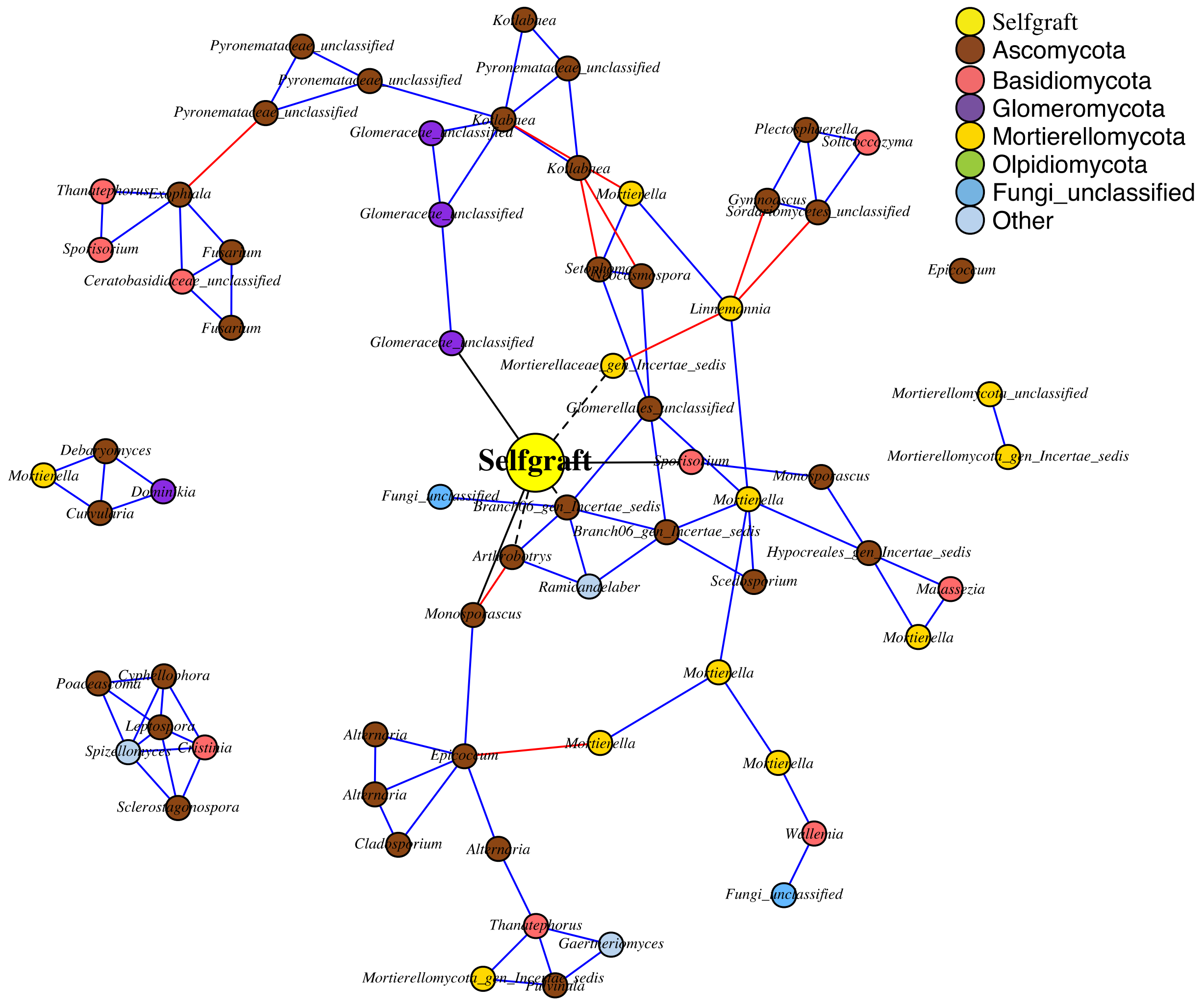
Selfgraft
Ascomycota
Basidiomycota
Glomeromycota
Mortierellomycota
Olpidiomycota
Fungi_unclassified
Other
Selfgraft
Kollabaea
Pyronemataceae_unclassified
Pyronemataceae_unclassified
Pyronemataceae_unclassified
Pyronemataceae_unclassified
Kollabaea
Glomeraceae_unclassified
Kollabaea
Glomeraceae_unclassified
Glomeraceae_unclassified
Plectosphaerella
Solicoccozyma
Gymnoascus
Sordariomycetes_unclassified
Mortierella
Thanatephorus
Exophiala
Sporisorium
Fusarium
Ceratobasidiaceae_unclassified
Fusarium
Setophoma
Pyrenochaetopsis
Epicoccum
Linnemannia
Mortierellaceae_gen_Incertae_sedis
Glomerellales_unclassified
Mortierellomycota_unclassified
Mortierellomycota_gen_Incertae_sedis
Debaryomyces
Mortierella
Dominikia
Curvularia
Sporisorium
Monosporascus
Fungi_unclassified
Branch06_gen_Incertae_sedis
Mortierella
Branch06_gen_Incertae_sedis
Arthrobotrys
Hypocreales_gen_Incertae_sedis
Ramicandelaber
Scedosporium
Malassezia
Monosporascus
Mortierella
Poaceascoma
Cyphellophora
Leptospora
Spizellomyces
Cristinia
Sclerostagonospora
Mortierella
Alternaria
Epicoccum
Mortierella
Alternaria
Mortierella
Cladosporium
Alternaria
Wallemia
Fungi_unclassified
Thanatephorus
Gaertneriomyces
Mortierellomycota_gen_Incertae_sedis
Pulvinula

B)

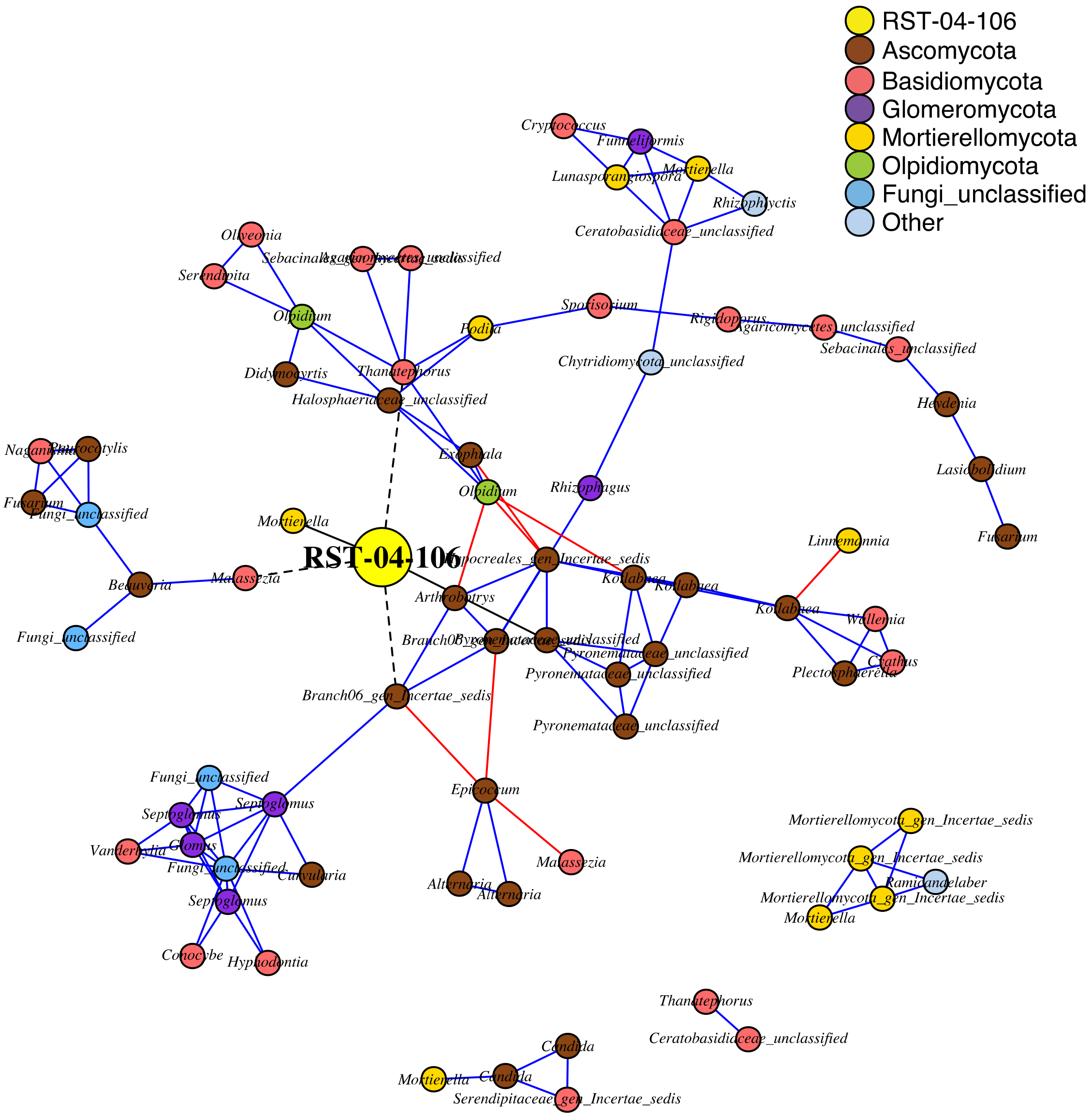
RST-04-106
Ascomycota
Basidiomycota
Glomeromycota
Mortierellomycota
Olpidiomycota
Fungi_unclassified
Other
RST-04-106
Cryptococcus
Funneliformis
Lunasporangiospora
Mortierella
Rhizophlyctis
Ceratobasidiaceae_unclassified
Oliveonia
Serendipita
Olpidium
Podila
Sporisorium
Rigidoporus
Sebacinales_unclassified
Chytridiomycota_unclassified
Didymocyrtis
Thanatephorus
Halosphaeriaceae_unclassified
Heydenia
Exophiala
Lasiobolidium
Rhizophagus
Fusarium
Fungi_unclassified
Beauveria
Malassezia
Linnemannia
Hypocreales_gen_Incertae_sedis
Kollarbea
Arthrobotrys
Wallemia
Pyronemataceae_unclassified
Cyathus
Plectosphaerella
Branch06_gen_Incertae_sedis
Epicoccum
Septoglomus
Vanderbylia
Glomus
Curvularia
Conocybe
Hyphodontia
Alternaria
Mortierellomycota_gen_Incertae_sedis
Ramicandelaber
Candida
Serendipitaceae_gen_Incertae_sedis

C)

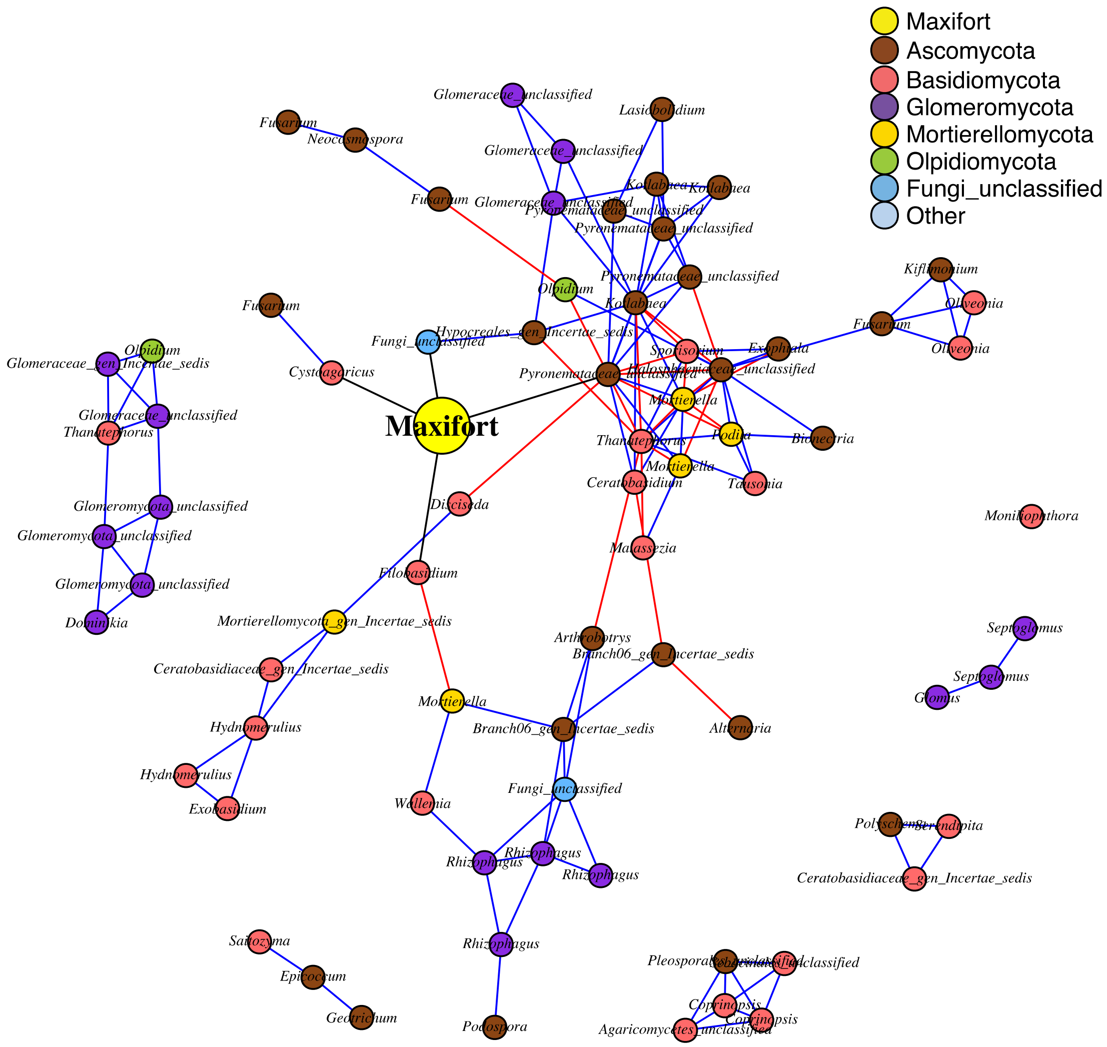

FIG S5 Phenotype-OTU network analysis (PhONA) of **endosphere** fungal taxa for tomato rootstock treatments: (A) selfgraft BHN589, (B) BHN589 grafted on RST-04-106, and (C) BHN589 grafted on Maxifort. Node color indicates the phylum, except that the yellow-color node represents yield associated with the rootstock. Nodes representing phyla with less than 1% relative abundance are labeled as ‘other’. Nodes connected to the rootstock yield node with black links are taxa that were predictive of rootstock yield, where dotted and solid lines indicate negative and positive associations with the yield node, respectively. Red and blue links represent

negative and positive associations, respectively, between OTUs. Nodes are labeled with the finest-resolution taxonomic categorization available.

A)

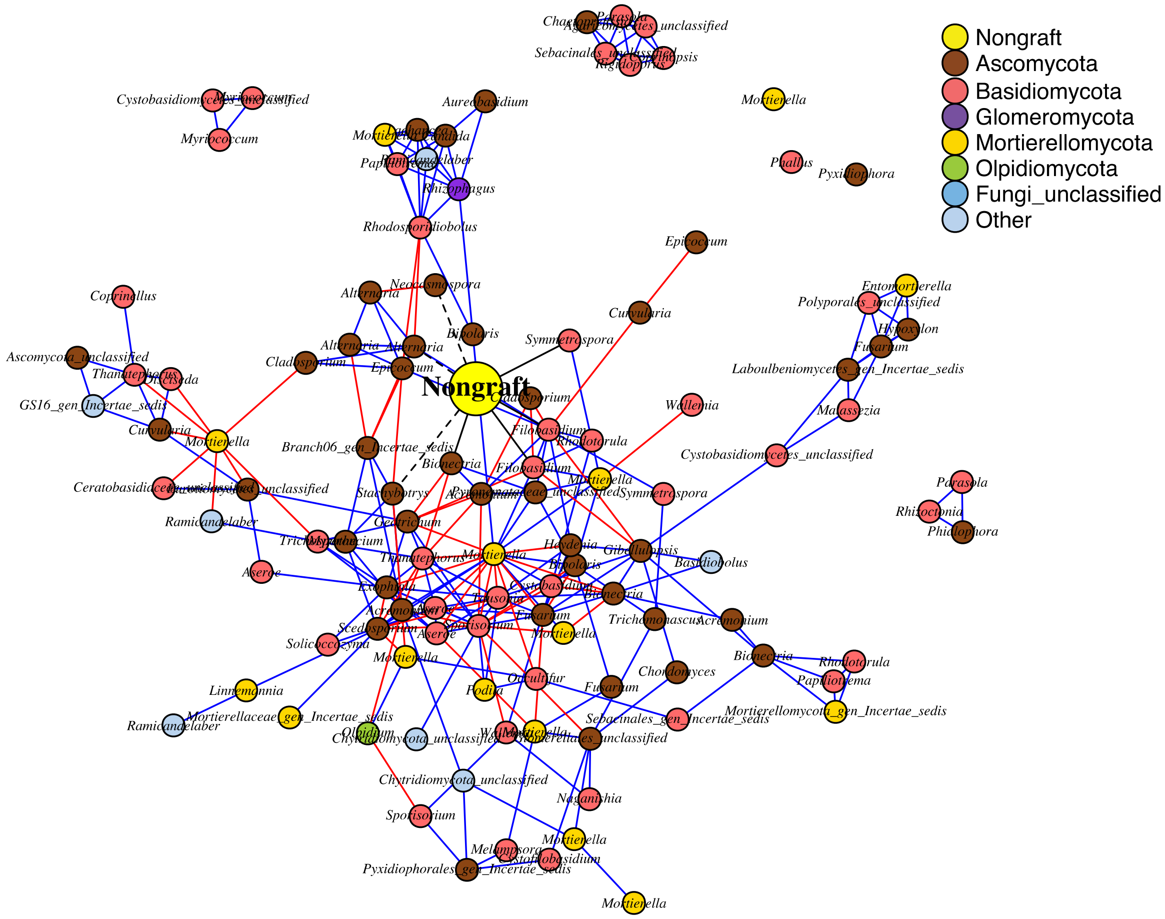
Nongraft
Ascomycota
Basidiomycota
Glomeromycota
Mortierellomycota
Olpidiomycota
Fungi_unclassified
Other
Nongraft
Aureobasidium
Rhizophagus
Rhodosporidiobolus
Epicoccum
Curvularia
Symmetrospora
Wallenia
Coprinellus
Ascomycota_unclassified
GS16_gen_Incertae_sedis
Myriococcum
Phallus
Pyxidiophora
Polyporales_unclassified
Laboulbeniomycetes_gen_Incertae_sedis
Malassezia
Cystobasidiomycetes_unclassified
Parasola
Rhizoctonia
Phialophora
Branch06_gen_Incertae_sedis
Stachybotrys
Geotrichum
Basidiobolus
Acremonium
Trichomonascus
Chordomyces
Linnemannia
Ramicandelaber
Mortierellaceae_gen_Incertae_sedis
Sebacinales_gen_Incertae_sedis
Mortierellomycota_gen_Incertae_sedis
Chytridiomycota_unclassified
Sporisorium
Naganishia
Pyxidiophorales_gen_Incertae_sedis
Mortierella

B)

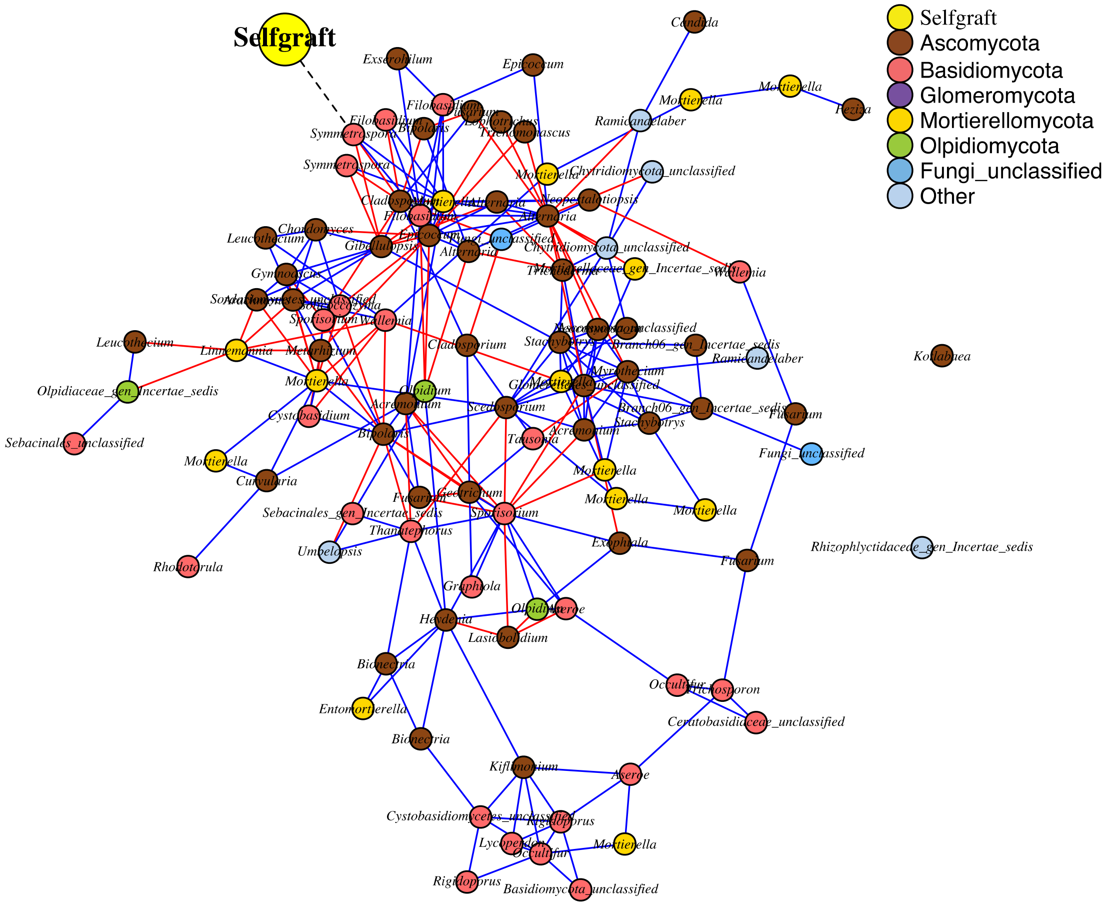
Selfgraft
Selfgraft
Ascomycota
Basidiomycota
Glomeromycota
Mortierellomycota
Olpidiomycota
Fungi_unclassified
Other

C)

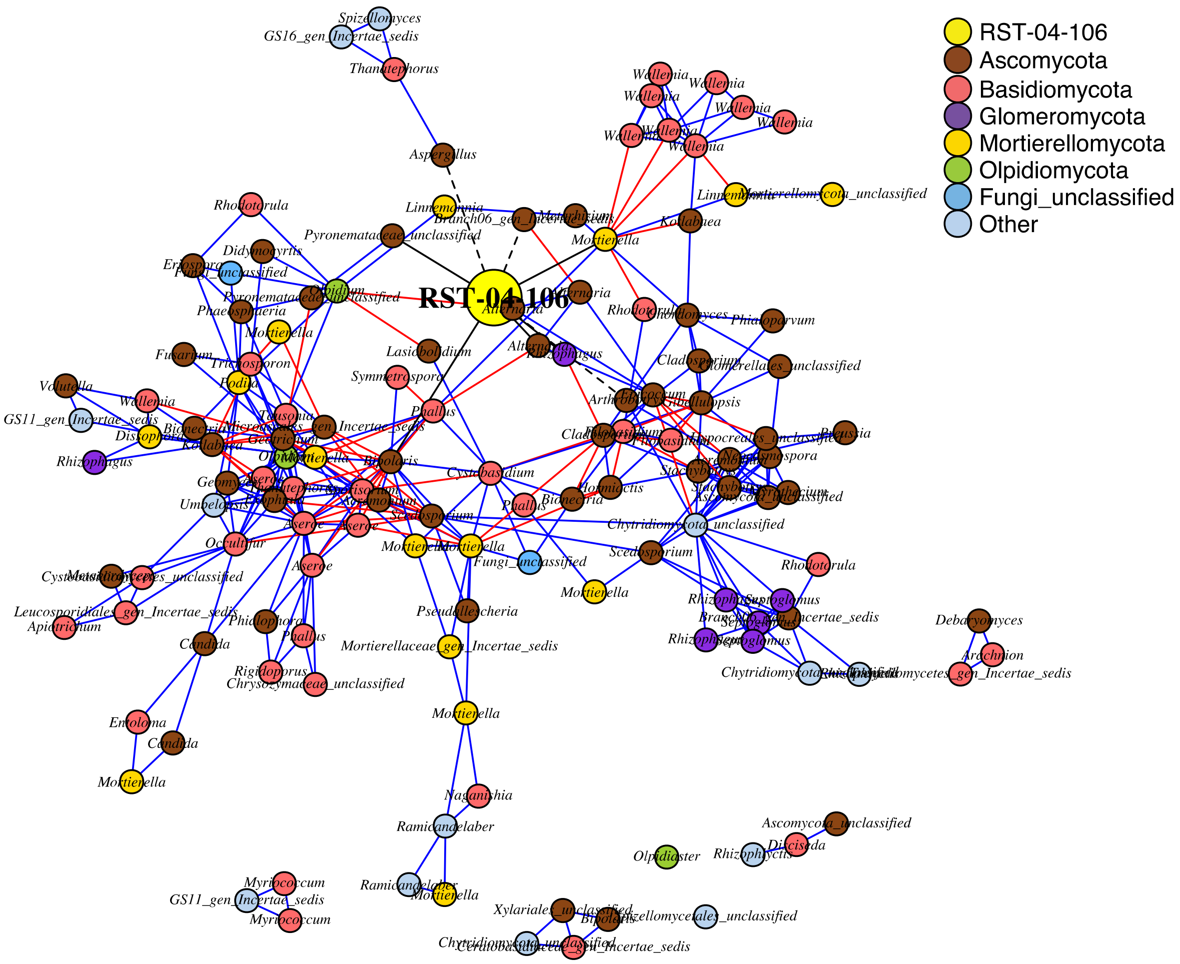
RST-04-106
Ascomycota
Basidiomycota
Glomeromycota
Mortierellomycota
Olpidiomycota
Fungi_unclassified
Other
RST-04-106

D)

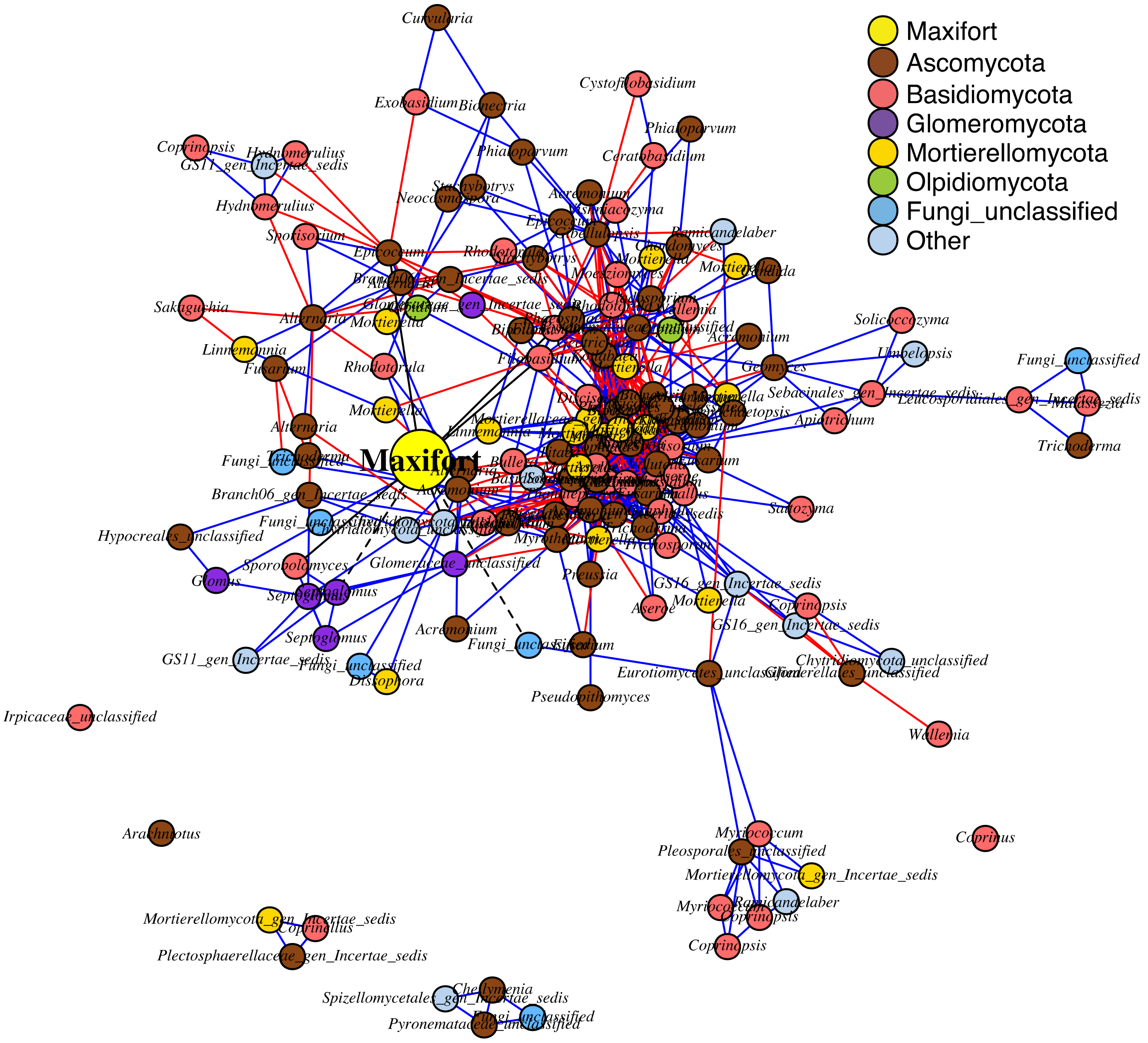

FIG S6 Phenotype-OTU network analysis (PhONA) of **rhizosphere** fungal taxa for tomato rootstock treatments: (A) nongraft and (B) selfgraft BHN589, and BHN589 grafted on two hybrid rootstocks ((C) RST-04-106 and (D) Maxifort). Node color indicates the phylum, except that the tomato-color node represents yield associated with the rootstock. Nodes representing phyla with less than 1% relative abundance are labeled as ‘other’. Nodes connected to the rootstock yield node with black links are taxa that were predictive of rootstock yield, where dotted and solid lines indicate negative and positive associations with the yield node,

respectively. Red and blue links represent negative and positive associations, respectively, between OTUs. Nodes are labeled with the finest-resolution taxonomic categorization available.

TABLE S1 Sites included in the study, their soil type, and geographic coordinates.

Study sites	Location	Latitude	Longitude	Soil type
Olathe Horticulture Research and Extension Center (OHREC)	Johnson County, KS	38.88N	94.99W	Chase silt loam
Common Harvest	Douglas County, KS	38.96N	95.20W	Eudora-Kimo complex

TABLE S2 Results of the multivariate permutational analysis of variance (PERMANOVA) for fungal taxon abundance data. Permutation was based on the Bray-Curtis distance matrix generated for root associated fungal communities at the OTU level from four tomato rootstock treatments: nongraft and selfgraft BHN589, and BHN589 grafted on two hybrid rootstocks (Maxifort and RST-04-106) (1000 permutations). P values < 0.05 are in bold.

**Factor**	**Sum of Squares**	**% Explained**	**P value**
Rootstock	1.22	2.05	< **0.05**
Compartment	5.47	9.17	< **0.001**
Study_site	5.04	8.44	< **0.001**
Year	3.18	5.33	< **0.001**
Rootstock:Compartment	0.58	0.97	0.972
Rootstock:Study_site	1.36	2.28	< **0.01**
Compartment:Study_site	1.60	2.68	< **0.001**
Rootstock:Year	0.95	1.59	0.1
Compartment:Year	0.99	1.66	< **0.001**
Study_site:Year	1.44	2.41	< **0.001**
Rootstock:Compartment:Study_site	0.53	0.89	0.998
Rootstock:Compartment:Year	0.48	0.80	0.998
Rootstock:Study_site:Year	1.18	1.98	< **0.05**
Compartment:Study_site:Year	0.60	1.01	< **0.05**
Rootstock:Compartment:Study_site:Year	0.60	1.00	0.989
Residual	34.46	57.75	
Total	59.68	100.00	
